# Editorial: Current Perspectives on Developmental Coordination Disorder (DCD)

**DOI:** 10.3389/fnhum.2022.837548

**Published:** 2022-02-14

**Authors:** Kate Wilmut, Jacqueline Williams, Catherine Purcell

**Affiliations:** ^1^Centre for Psychological Research, Oxford Brookes University, Oxford, United Kingdom; ^2^Institute for Health and Sport, College of Sport and Exercise Science, Victoria University, Melbourne, VIC, Australia; ^3^School of Healthcare Sciences, College of Biomedical and Life Sciences, Cardiff University, Cardiff, United Kingdom

**Keywords:** developmental coordination disorder, DCD, co-occurring disorders, genome, motor skill, intervention—behavioral

## Introduction

Developmental Coordination Disorder (DCD) occurs in approximately 5% of children (Blank et al., 2019) and describes a condition in which motor coordination is below the level expected given a child's age and opportunity for learning (APA, 2013). Children with DCD display motor difficulties which persist into adulthood and cannot be better explained by a medical or neurological condition (APA, 2013). The difficulties that individuals with DCD experience have a significant impact on activities of daily living, scholastic achievement, inter-personal relationships, and employment (Kirby et al., 2010). In addition, secondary consequences of DCD include higher anxiety (Harris et al., 2021), poorer levels of physical fitness (Schott et al., 2007) and negative self-perceptions (Piek et al., 2006). Despite significant growth in research into DCD over the last four decades, and international clinical practice guidelines being released (Blank et al., 2019), there are still pending questions regarding etiology, the influences of co-occurrence, movement behavior and ways in which change can be promoted. This Research Topic aimed to capture the breadth of the recent focus of research into DCD.

## Etiology

Mountford et al. presented a genome-wide association study to examine potential biological drivers of early motor coordination in the context of motor difficulties. The authors looked for common genetic variants (single nucleotide polymorphisms) which could explain poor motor coordination and identified 59 genetic variants within five genes. Although some caution is necessary, as the inclusion of data was based solely on motor function rather than all of the DSM-5 criteria, this study is an exciting additional insight into the potential genes which may drive DCD.

## Co-Occurrence

Two studies within this topic considered co-occurrence. Meachon et al. considered inhibitory function in adults with ADHD, with DCD or with combined ADHD and DCD. Behaviourally the groups did not differ and performed as well as age-matched peers. However, event related potentials measured using EEG differed among the study groups and typical controls. In addition, differences were identified across co-occurrence groups. These data point toward compensatory mechanisms in simple tasks, resulting in typical behavior even in the face of atypical underlying mechanisms, with the latter differing among diagnostic subgroups. Izadi-Najafabadi and Zwicker used MRI to consider white matter microstructure before and after intervention using Cognitive Orientation to Occupational Performance (CO-OP) in children with and without co-occurring ADHD. Children with DCD showed significant alterations in white matter microstructure. In contrast, the children with DCD and ADHD showed no such white matter changes despite intervention improving motor performance. This suggests CO-OP has a distinctly different neurological effect on children with DCD and ADHD compared to those just with DCD and that some modifications to the CO-OP protocol may better address the needs of children with DCD and ADHD. Together these studies emphasize that the neurology underpinning behavior in those with and without co-occurrences can be very different.

## Measured Behavior

When asked to perform a stepping task, Parr et al. found that children with DCD were more variable in their foot placement compared to children without DCD. However, neither gaze behavior nor state-anxiety differed across groups. In a second study, Parr et al. examined stair ascent and descent finding again that variability was higher in children with DCD and that these children looked further ahead than their typically developing peers, these factors may increase the likelihood of a fall. Children with DCD also reported significantly higher state-anxiety prior to stair descent. Switching to a manual task, Krajenbrink et al. considered motor planning within a grasping task, building on a plethora of research in the last 5 years which has raised the question of whether children with DCD *choose* not to or *cannot* plan for comfort. These authors used an established sword-task and a newly developed hammer-task. Differences between the two tasks led the authors to suggest that children with DCD can plan for comfort when it is demanded by task constraints. Finally, Blais et al. considered whether a specific auditory timing deficit contributes to DCD by exploring if children with DCD can synchronize motor output to sensory stimuli. Their data suggested a potential deficit in timing perception more generally in children with DCD, but that the learning of temporal motor sequences may be improved in DCD with the use of visual cues.

Three studies considered the experiences and abilities of adults with DCD on the road. Gentle et al. used a driving simulator to consider driving characteristics under three conditions of increasing complexity. The data suggest that young adults with DCD behave differently to their peers when driving in progressively more complex environments. Warlop et al. considered the hazard perception of cyclists with and without DCD and found that although eye fixation on hazards was significantly different among groups, no differences were observed in terms of reaction speed or identification of hazards. To explore the potential vulnerability of individuals with DCD as pedestrians, Wilmut and Purcell asked parents of children with DCD and adults with DCD about their roadside experiences. Individuals with DCD alongside ADHD/ASD were significantly more likely to engage in risky looking behaviors. Regardless of co-occurrence the vast majority of participants reported that motor difficulties influenced road crossing behavior. Although these studies are all very different, taken together they demonstrate clear difference between those with and those without DCD in high risk situations.

All of these studies are a timely reminder that the emerging movement we observe is a consequence of the *individual* we are measuring, the *task* we have set and the *environment* we have set up (Newell, 1986).

## Promoting Change

Finally, Smits-Engelsman et al. considered whether exergames could be used to improve physical fitness in children with DCD. A 10-week intervention improved both aerobic and anaerobic fitness as well as balance and coordination in children with DCD and controls. The findings highlight the potential for these games to do more than improve motor control and given the ease with which families can engage in exergames, without sustained input from a healthcare professional, their potential to be an affordable and accessible intervention is promising. In another study, Grohs et al. considered whether transcranial direct current stimulation (tDCS) of the primary motor cortex could boost motor learning. Although motor skill improved over the 5-day learning period, motor cortex tDCS did not enhance motor learning. The authors concede that targeting the cerebellum may have produced a different result. This is the first study to examine the therapeutic efficacy of tDCS on motor learning in children with DCD which future studies can draw upon. Again, although these are very different they highlight potential new avenues for how motor control can be influenced and changed.

## And Finally……

Included in this topic was a paper by Scott et al. which sits within Frontiers for Young Minds. This paper is not addressed to the scientific community but rather explains motor imagery and action observation interventions for children with DCD. Given the inaccessibility of academic research for certain stakeholders this is an excellent example of how scientific research can be disseminated to young people.

## Conclusions

This Research Topic represents the reality of the broad range of diverse research being undertaken within the field of DCD. Within the DCD topic we see exciting progress in terms of understanding the underlying biology which is driving the behavior which we are all so familiar with. However, this is complemented by research aiming at understanding the individual experience of DCD and the impact it has on activities of daily life such as driving, crossing the road and walking down the stairs. This is a far cry from the very lab based work of 10 years ago. Finally, we also see work focusing on novel interventions and how we can help ensure movement is functional for people with DCD and work modeling how our findings can be disseminated to a wide audience.

## Author Contributions

All authors listed have made a substantial, direct, and intellectual contribution to the work and approved it for publication.

## Conflict of Interest

The authors declare that the research was conducted in the absence of any commercial or financial relationships that could be construed as a potential conflict of interest.

## Publisher's Note

All claims expressed in this article are solely those of the authors and do not necessarily represent those of their affiliated organizations, or those of the publisher, the editors and the reviewers. Any product that may be evaluated in this article, or claim that may be made by its manufacturer, is not guaranteed or endorsed by the publisher.
